# Culturable nitrogen-transforming bacteria from sequential sedimentation biofiltration systems and their potential for nutrient removal in urban polluted rivers

**DOI:** 10.1038/s41598-021-86212-3

**Published:** 2021-04-02

**Authors:** Arnoldo Font Nájera, Liliana Serwecińska, Joanna Mankiewicz-Boczek

**Affiliations:** 1 UNESCO Chair On Ecohydrology and Applied Ecology, Faculty of Biology and Environmental Protection, University of Lodz, Banacha 12/16, 90-237 Łódź, Poland; 2 European Regional Centre for Ecohydrology of the Polish Academy of Sciences, Tylna 3, 90-364 Łódź, Poland

**Keywords:** Environmental biotechnology, Bacteria, Environmental microbiology

## Abstract

Novel heterotrophic bacterial strains—Bzr02 and Str21, effective in nitrogen transformation, were isolated from sequential sedimentation-biofiltration systems (SSBSs). Bzr02, identified as *Citrobacter freundii*, removed up to 99.0% of N–NH_4_ and 70.2% of N–NO_3_, while Str21, identified as *Pseudomonas mandelii*, removed up to 98.9% of N–NH_4_ and 87.7% of N–NO_3_. The key functional genes *nap*A/*nar*G and *hao* were detected for Bzr02, confirming its ability to reduce nitrate to nitrite and remove hydroxylamine. Str21 was detected with the genes *nar*G, *nir*S, *nor*B and *nos*Z, confirming its potential for complete denitrification process. Nitrogen total balance experiments determined that Bzr02 and Str21 incorporated nitrogen into cell biomass (up to 94.7% and 74.7%, respectively), suggesting that nitrogen assimilation was also an important process occurring simultaneously with denitrification. Based on these results, both strains are suitable candidates for improving nutrient removal efficiencies in nature-based solutions such as SSBSs.

## Introduction

The excessive inflow of nitrogen compounds has been a serious problem for water bodies in urban areas, including rivers and ponds. High concentrations of NH_4_^+^, NO_3_^−^ and NO_2_^−^ contribute to the occurrence of favourable conditions for the proliferation of phytoplankton, including cyanobacteria, which consequently affect aquatic and human health with the production of toxins, the decrease of light penetration and the depletion of oxygen in the pelagic zone^1–3^. To address the above-mentioned problem in urban polluted rivers, sequential sedimentation-biofiltration systems (SSBSs) have been implemented. These systems are designed according to the principles of ecohydrology to enhance the capacity of natural systems to remove environmental pollutants and are considered as nature-based solutions (NBS)^4,5^. These eco-friendly systems use a combination of natural processes for water treatment, i.e., sedimentation of solids, absorption of phosphorus, reduction of excessive nitrogen compounds by stimulating denitrification and nitrification processes and phytoremediation. SSBSs are constructed upstream of ponds or reservoirs to reduce anthropogenic eutrophication and, among others, the development of harmful algal blooms including toxic cyanobacteria. These systems have been observed to remove nitrogen compounds up to 59.8% of NH_4_^+^, 55% of NO_2_^−^, 91.3% of NO_3_^−^ and 56.9% of total nitrogen (TN)^6–9^. The protection of urban ponds is needed because they regulate water flow and soil erosion during storms, increase the water retention, provide humidity, promote plant evapotranspiration and influence the cooling of urban areas. Moreover, urban ponds also offer aesthetic value, environmental education and recreational opportunities^10–12^.

The important elements for the effective functioning of SSBSs are the structure and metabolic activity of microorganism inhabiting sediments. Microbial communities, with special consideration on bacteria, have been recently studied in working SSBSs^9^. The significant positive correlations observed between the measured concentration of nutrients (NO_3_^−^ and NH_4_^+^) and the abundance of bacterial genes involved in nitrification and denitrification processes indicated that bacterial communities have played an important role in nitrogen transformations. Therefore, in the present study we focused on the characteristics of isolated bacterial strains capable of nitrogen removal.

The nitrification involves two consecutive reactions (NH_4_^+^ → NO_2_^−^ → NO_3_^−^), and it has been studied in different autotrophic strains: (i) the first reaction was described in ammonia oxidizing bacteria (AOB), in the genera *Nitrosomonas*, *Nitrosospira* (β-Proteobacteria) and *Nitrosococcus* (ϒ-Proteobacteria)^13,14^; while (ii) the second reaction in nitrite oxidizing bacteria (NOB), in the genera *Nitrobacter* (α-proteobacteria), *Nitrococcus* (ϒ-Proteobacteria) and *Nitrospina*^15^*.* Nitrification also occurs in direct oxidation of NH_4_^+^ → NO_3_^−^ (complete ammonium oxidation, COMAMMOX) by autotrophic strains of *Nitrospira* spp. (Class Nitrospirae)^16,17^. Moreover, nitrification via the hydroxylamine (NH_2_OH) pathway, which is an intermediary product between the first nitrification reaction (ammonia oxidation to hydroxylamine), has also been described for *Nitrosomonas*^18^ and heterotrophic strains of *Acinetobacter*^19^, *Janthinobacterium*^20^, *Alcaligenes*^21^, *Enterobacter*^22^, and *Pseudomonas*^23–25^. Denitrification—a dissimilatory nitrate reduction (DNR) pathway—involves four cascade reactions for the transformation of NO_3_^−^ → NO_2_^−^ → NO → N_2_O → N_2_, which was initially described for heterotrophic facultative anaerobic bacterial strains^26,27^. More recently, research has been focused in the identification of aerobic denitrifying strains that can perform parallel nitrification due to their potential utilization in waste water treatment plants (WWTPs) for the complete removal of nitrogen compounds. Several strains have been isolated and reported to perform simultaneous nitrification–denitrification (SNdN), with the most common genera represented by *Acinetobacter, Agrobacterium, Alcaligenes, Bacillus*, *Klebsiella*, *Enterobacter* and *Pseudomonas*^28^.

The majority of the above described nitrogen transforming bacteria have been isolated from sewage in WWTPs, constructed wetlands (CWs) or biofilm formations in experimental bioreactors^28^. To our knowledge, the bacteria carrying out nitrogen transformation processes have not yet been isolated and characterized within the SSBSs. Additionally, there is a limited number of studies discussing the nitrogen balance, most of which were in controlled experiments for selected bacterial strains, in order to confirm their preferred metabolic pathways^29–33^.

Therefore, the present study aimed to isolate and characterize heterotrophic bacterial strains that naturally occur in SSBSs, which are responsible for nitrogen transformation in nitrification and denitrification processes. To reach the objective, culturable bacteria were isolated from sediments, nitrogen transformation pathways were determined, and nitrogen balance was described. Additionally, the preference of the strains to perform nitrogen assimilatory over dissimilatory transformation processes was also investigated. Our results were compared with the nitrogen removal efficiency of other published isolated bacterial strains and discussed in the context of biotechnological potential of selected strains to improve the nutrient removal efficiency in NBS technologies.

## Results and discussion

### Selection and identification of potential nitrogen transforming bacteria

#### Initial screening of bacteria capable of nitrogen utilization

Ten bacterial strains were selected for their ability to transform nitrogen compounds and were summarized in Table 1. All mentioned strains were able to utilize NO_3_^−^ in Giltay denitrifying medium (GiDM). Seven strains (Str21, Bzr07, Sok01, Sok03, Sok06, Sok20 and Sok41) presented no accumulation of NO_2_^−^, suggesting that it was further reduced by bacteria (Table 1). In contrast, three strains (Bzr02, Str01 and Sok05), only transformed NO_3_^−^ to NO_2_^−^, which was then accumulated in the medium with no further utilization (Table 1).

**Table 1:** Bacterial strains capable of nitrogen transformation in different media.

No	Strain	Microbiological analysis							
		Denitrification medium (DM)			Nitrification medium (NM)				
		GiDM	Gas formation	GNM	SNM	CNM	ANM	GNM + NH_2_OH	
		Glucose + NO_3_ −		Glucose + NH_4_ +	Succinate + NH_4_ +	Citrate + NH_4_ +	Acetate + NH_4_ +	Glucose + NH_2_OH + NH_4_ +	
1	Str21	−NO_2_ − (24 h)	+	−NH_4_ + (24 h)	−NH_4_ + (72 h)	−NH_4_ + (72 h)	NG	NG	
2	Bzr02	+ NO_2_ − (48 h)	−	−NH_4_ + (24 h)	−NH_4_ + (72 h)	−NH_4_ + (72 h)	−NH_4_ + (144 h)	−NH_4_ + (72 h)	
3	Bzr07	−NO_2_ − (24 h)	−	−NH_4_ + (24 h)	−NH_4_ + (72 h)	+NH_4_ + (72 h)	NG	NG	
4	Str01	+NO_2_ − (48 h)	−	NG	−NH_4_ + (72 h)	−NH_4_ + (72 h)	−NH_4_ + (144 h)	NG	
5	Sok03	−NO_2_ − (24 h)	−	NG	−NH_4_ + (72 h)	−NH_4_ + (72 h)	NG	NG	
6	Sok05	+NO_2_ − (48 h)	−	−NH_4_ + (120 h)	NG	NG	−NH_4_ + (144 h)	NG	
7	Sok41	−NO_2_ − (24 h)	+	−NH_4_ + (24 h)	−NH_4_ + (72 h)	NG	NG	NG	
8	Sok01	−NO_2_ − (24 h)	+	NG	NG	NG	NG	NG	
9	Sok06	−NO_2_ − (24 h)	+	NG	NG	NG	NG	NG	
10	Sok20	−NO_2_ − (24 h)	+	NG	NG	NG	NG	NG	

No	Strain	Genetic analysis							
		Taxonomy	Assimilation	Nitrification			Denitrification		
		16S r RNA	*nas*A	*hao*	*nap*A/*nar*G		*nir*S	*nor*B	*nos*Z
			NO_3_ − → NO_2_ −	NH_2_OH → NO_2_ −	NO_3_ − → NO_2_ −		NO_2_ − → NO	NO → N_2_O	N_2_O → N_2_
1	Str21	*Pseudomonas mandelii*^a^	+ ^c^	−	−	+ ^c^	+ ^c^	+ ^c^	+ ^c^
2	Bzr02	*Citrobacter freundii*^b^	−	+ ^c^	+ ^c^	+ ^c^	−	−	−
3	Bzr07	*Pseudomonas migulae*	−	−	+	−	−	−	+
4	Str01	*Bacillus simplex*	−	−	−	+	−	−	−
5	Sok03	*Pseudomonas guineae*	−	−	−	−	−	−	+
6	Sok05	*Kocuria rosea*	−	−	−	+	−	−	−
7	Sok41	*Acidovorax radicis*	−	−	−	+	−	−	+
8	Sok01	*Hydrogenophaga taeniospiralis*	−	−	−	+	−	−	+
9	Sok06	*Bacillus aereus*	−	−	−	+	−	−	+
10	Sok20	*Janthinobacterium lividum*	−	−	−	+	−	−	+

- NO_2_^−^: no detection or full transformation of nitrite; + NO_2_^−^: detection or incomplete transformation of nitrite; (#h): incubation hours; + : detection; −: no detection;—NH_4_^+^: no detection or full transformation of ammonium; + NH_4_^+^: detection or incomplete transformation of ammonium; NG: no bacterial growth. Taxonomical ID for the strain Str21^a^ was also confirmed with the sequence analysis of *rpo*B gene in supplementary **Fig S1**, and the Strain Bzr02^b^ with BIOLOG Gen III plates in supplementary **Table S2**. ^c^ The nucleotide BLAST similarity analysis for the functional genes detected in strains Str21 and Bzr02 was presented in supplementary **Table S3.**

In turn, seven among 10 selected strains (Str21, Bzr02, Bzr07, Str01, Sok03, Sok05 and Sok41) were able to utilize NH_4_^+^ on various nitrifying media with different carbon sources (Table 1). The most efficient removal of NH_4_^+^ was found in nitrifying medium containing glucose—GNM (up to 48 h for the strains Str21, Bzr02, Bzr07, Sok05 and Sok41; Table 1).

#### Taxonomic and phylogenetic characteristics

**Figure 1:**
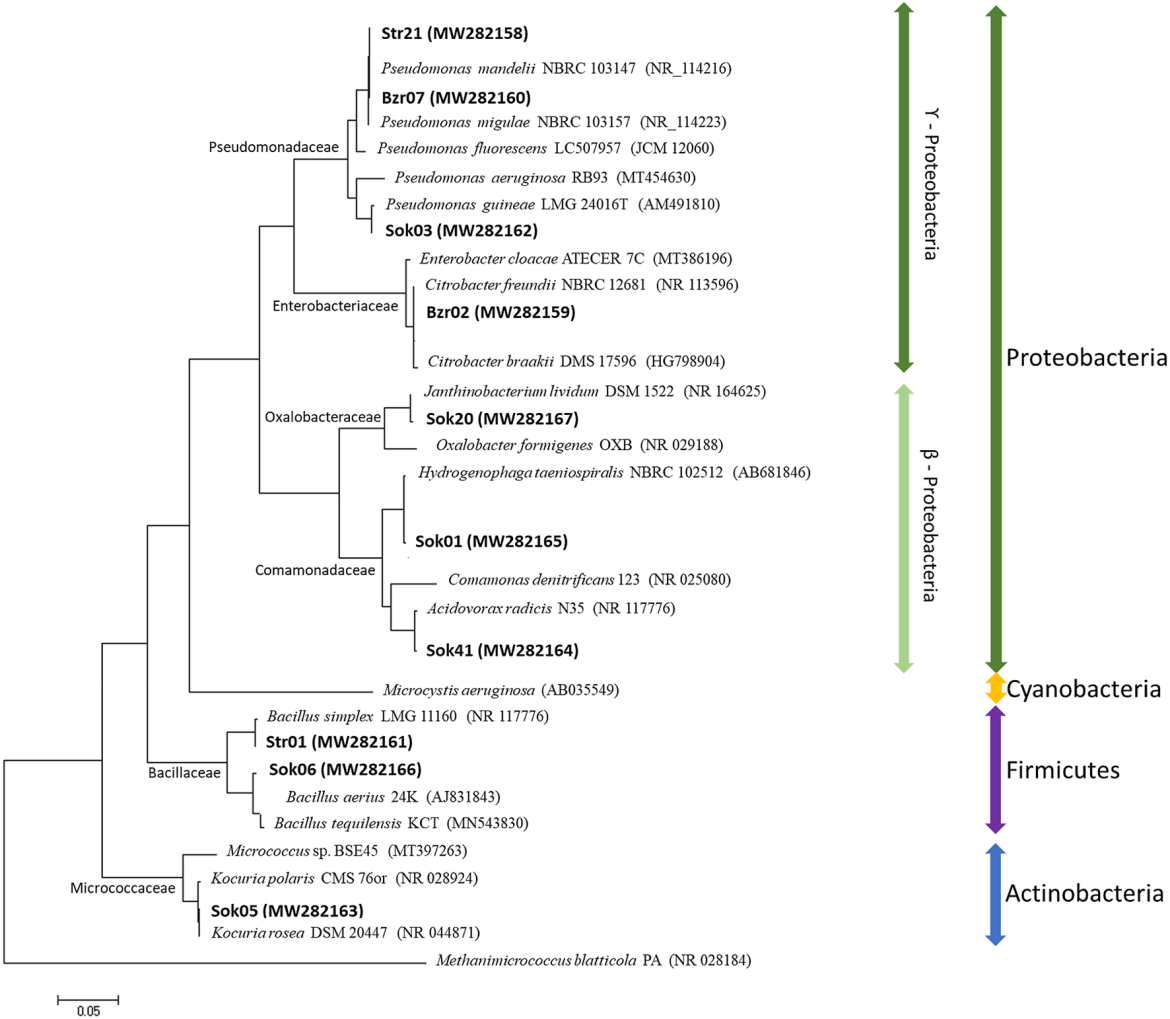
Neighbour-joining phylogenetic tree construction for the nitrogen transforming bacteria isolated in SSBSs. The tree was constructed using the 16S rRNA sequences obtained from GenBank (accession number inside the brackets). The bar under the graph represents the nucleotide substitutions per position. The sequence of *Microcystis aeruginosa* was used as an outgroup to cluster the representative strains in the phylum Proteobacteria, and the sequence of *Methanimicrococcus blatticola* PA (Archaea) as an outgroup to cluster the different bacteria phyla.

Taxonomical characteristics of selected bacterial isolates, based on the 16 s rRNA, were presented in Table 1, and their phylogenetic relationships were described in Fig. 1. The sequence homology revealed that the studied bacteria belong to significantly different taxonomical groups (Supplementary Table S1). Seven of them were clustered within the phylum Proteobacteria but different bacterial families: (i) the strains Str21, Bzr07 and Sok03, within the family Pseudomonadaceae, presented high similarity with *Pseudomonas mandelii* (99.55%), *P. migulae* (99.83%) and *P. guineae* (99.45%), respectively, (ii) the Sok01 was similar to *Hydrogenophaga taeniospiralis* (99.32%) and the Sok41 to *Acidovorax radicis* (99.29%), both strains within the family Comamonadaceae, (iii) the Bzr02 was similar to *Citrobacter freundii* (99.39%), which belongs to the family Enterobacteriaceae, and (iv) the strain Sok20 to *Janthinobacterium lividum* (99.34%), which belongs to the family Oxalobacteriaceae (Fig. 1). Furthermore, the strains Str01 and Sok06, within the phylum Firmicutes, presented high similarity to *Bacillus simplex* (98.36%) and *B. aereus* (99.63%)*,* respectively, and the strain Sok05 to *Kocuria rosea* (99.08%) in the phylum Actinobacteria (Fig. 1).

#### Proposed metabolic pathways for nitrogen transformation

Possible bacterial metabolic pathways for nitrogen transformation were described based on the amplification of key functional genes involved in the nitrogen cycling process (Table 1 and supplementary Fig S2). The strains Str01 and Sok05 were considered to be nitrate reducers, since NO_2_^−^ was accumulated in GiDM (Table 1). The above suggestion was supported with the detection of the *nar*G gene (respiratory nitrate reductase), which is involved in the reduction of NO_3_^−^ → NO_2_^−^ in anaerobic conditions (Table 1 and supplementary Fig S2). The strains Sok01, Sok06, and Sok20 were considered to be facultative anaerobic denitrifiers, since they were able to continue the reduction of NO_3_^−^ to gas in GiDM, but could not utilize NH_4_^+^ in any of the nitrifying media in aerobic conditions (Table 1 and supplementary Fig S2). In contrast, the strains Sok41, Sok03 and Bzr07 were considered to be facultative anaerobic denitrifiers that could also utilize NH_4_^+^ in aerobic conditions (Table 1). All six facultative anaerobic denitrifiers (Sok01, Sok41, Sok20, Sok06, Sok03 and Bzr07) presented the *nos*Z gene (Table 1), which is involved in the last step of denitrification, and therefore, suggested that they performed complete reduction of NO_3_^−^ → N_2_.

Bzr02 (*Citrobacter freundii*) and Str21 (*Pseudomonas mandelii*), isolated from the Bzr-SSBS and Str-SSBS, respectively, presented the best results during the screening experiments on transformation of nitrogen compounds. Both strains were able to grow and remove NO_3_^−^ and NH_4_^+^ in a lower time of incubation in different culture media, and were observed with the highest number of studied key functional genes involved in assimilation, nitrification or denitrification processes (Table 1). Moreover, the Bzr02 was the only strain capable to utilize NH_4_^+^ with the presence of hydroxylamine in GNM, suggesting that hydroxylamine could be an intermediary product in the nitrification process. Therefore, Bz02 and Str21 were selected for further quantitative experiments in nitrogen transformation assays.

### Nitrogen transforming processes—strains Bzr02 and Str21

#### Ammonium transformation in nitrifying medium

**Figure 2:**
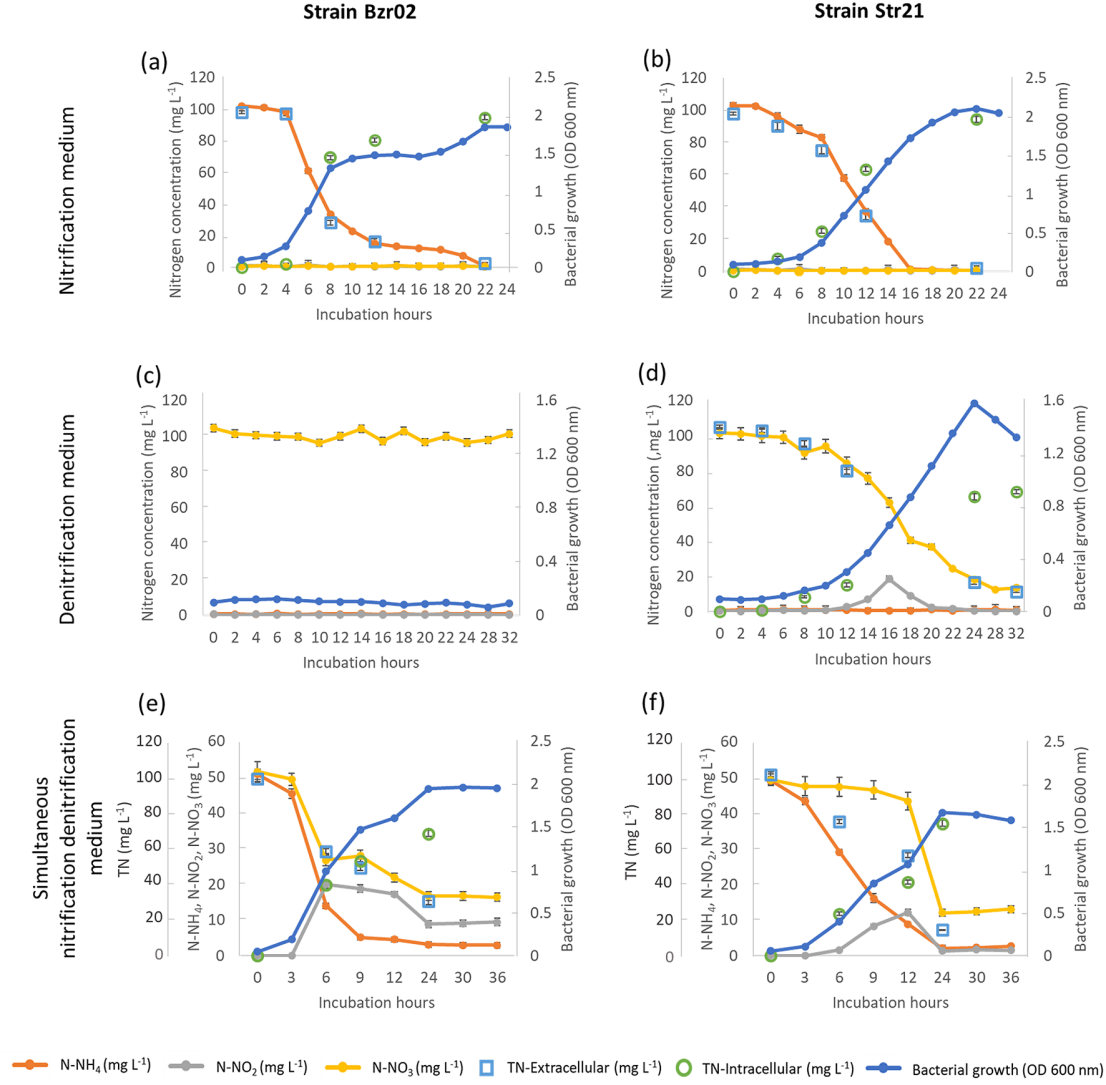
Dynamics of nitrogen transformation for strains Bzr02 and Str21 in nitrifying medium NM (**a**, **b**, respectively), denitrifying medium DM (**c**, **d**, respectively) and simultaneous nitrifying-denitrifying medium SNDM (**e**, **f**, respectively). Values represent the mean and the standard error (n = 3).

Bzr02 and Str21 were cultivated in nitrifying medium (NM) under aerobic conditions, and their growth and utilization of N–NH_4_ were followed for 24 h (Fig. 2a,b). The average and maximum removal rates of N–NH_4_ for both strains were described in Table 2. Both strains were able to utilize N–NH_4_ as a sole nitrogen source. Bzr02 presented a 4 h lag phase with minimal growth at the beginning of the assay (Fig. 2a). The log phase was observed after 4 h of incubation (Fig. 2a), which correlated with the maximum removal rate of N–NH_4_ (16.17 ± 0.97 mg L^−1^ h^−1^, Table 2). A stationary phase occurred between 12 and 18 h, however, the strain was able to remove 82.6% of N–NH_4_ until 14 h of incubation (Fig. 2a). The maximum removal of N–NH_4_ was observed at 22 h of incubation (99.0 ± 0.2%; Table 2). The average removal rate of N–NH_4_ was 5.41 ± 0.13 mg L^−1^ h^−1^ (Table 2), which was significantly higher from other published strains: *Alcaligenes denitrificans* WY200811 (0.69 mg L^−1^ h^−1^)^34^, *Klebsiella pneumonae* EGD-HP19-C (2.29 mg L^−1^ h^−1^)^35^, *K. pneumonae* CF-S9 (4.3 mg L^−1^ h^−1^)^36^ and *Enterobacter cloacae* CF-S27 (2.22 mg L^−1^ h^−1^)^22^.

**Table 2:** Nitrogen removal rates by strains Bzr02 and Str21 in different nitrogen media.

Medium	Nitrogen source	Bzr02			Str21		
		Average (mg L^−1^ h^−1^)	Maximal (mg L^−1^ h^−1^)	Removal (%)	Average (mg L^−1^ h^−1^)	Maximal (mg L^−1^ h^−1^)	Removal (%)
NM	N-NH_4_	5.41 ± 0.13	16.17 ± 0.97	99.0 ± 0.2	7.21 ± 0.12	10.20 ± 0.25	98.9 ± 0.6
DM	N-NO_3_	NT	NT	NT	3.89 ± 0.16	6.66 ± 0.27	87.7 ± 0.2
SNDM	N-NH_4_	5.07 ± 0.09	10.44 ± 0.18	94.1 ± 1.3	3.35 ± 0.04	4.52 ± 0.22	95.6 ± 1.5
	N-NO_3_	1.44 ± 0.16	7.52 ± 0.10	70.2 ± 3.6	2.29 ± 0.22	2.61 ± 0.17	75.4 ± 2.6

NM: nitrifying medium; DM: denitrifying medium; SNDM: simultaneous nitrifying-denitrifying medium; NT: Not transformed. Values represent the mean and the standard error (n = 3).

Str21 presented a 6 h lag phase, however, utilization of N–NH_4_ started after 2 h of incubation (Fig. 2b). The maximum removal rate of N–NH_4_ was observed after 8 h (10.2 ± 0.25 mg L^−1^ h^−1^; Table 2), which continued until almost complete depletion under 16 h of incubation (98.9 ± 0.6%; Table 2). The average removal rate of N–NH_4_ was 7.21 ± 0.12 mg L^−1^ h^−1^ (Table 2), which was significantly higher from other strains in the family Pseudomonadaceae: *Pseudomonas* sp. JQ-H3 (2.7 mg L^−1^ h^−1^)^33^, *P. stutzeri* YZN-001 (5.53 mg L^−1^ h^−1^)^37^, *P. stutzeri* AD1 (3.1 mg L^−1^ h^−1^)^38^, *P. tolaasii* Y-11 (2.04 mg L^−1^ h^−1^)^39^, and similar to *P. putida* Y-9 (7.4 mg L^−1^ h^−1^)^24^ and *P. stutzeri* T13 (7.09 mg L^−1^ h^−1^)^30^.

The concentrations of N–NO_2_ and N–NO_3_ were insignificant through the complete assays for Br02 and Str21, and therefore no nitrification products were observed to occur (Fig. 2a,b, respectively). Similar results were published for all the above-mentioned strains and other genera, i.e., *Bacillus* SB1^40^ and *Acinetobacter* sp. SYF26^41^.

#### Nitrate transformation in denitrifying medium

Bzr02 and Str21 were cultivated in denitrifying medium (DM) under aerobic conditions, and their growth and utilization of N–NO_3_ were followed for 32 h (Fig. 2c,d). The average and maximum removal rates of N–NO_3_ for both strains were described in Table 2. Bzr02 was not able to grow and transform N–NO_3_ when it was added to the medium as the sole nitrogen source. Similar results were reported for *Acinetobacter calcoaceticus* HNR^19^, and it was proposed that the strain was sensitive to an initial high concentration of N–NO_3_ (40 mg L^−1^) in denitrifying medium. The above observation suggests that Bzr02 was also sensitive to the high initial concentration of N–NO_3_ (100 mg L^−1^) in DM.

On the contrary, Str21 was able to utilize N–NO_3_ as a sole nitrogen source in DM (Fig. 2d). After a 6 h lag phase, the strain began to grow until the log phase was observed from 12 h of incubation (Fig. 2d). The maximum removal rate of N–NO_3_ was 6.66 ± 0.27 mg L^−1^ h^−1^ (Table 2). Str21 removed N–NO_3_ to a maximum of 87.7 ± 0.16% during 28 h of incubation. The average removal rate of N–NO_3_ was 3.89 ± 0.27 mg L^−1^ h^−1^, which was significantly higher than other strains in the family Pseudomonadaceae: *Pseudomonas* sp. JQ-H3 (1.78 mg L^−1^ h^−1^)^33^, *P. tolaasii* Y-11 (2.04 mg L^−1^ h^−1^)^39^ and *P. stutzeri* AD1 (1.98 mg L^−1^ h^−1^)^38^, and other bacteria: *Klebsiella pneumonae* CF-S9 (2.2 mg L^−1^ h^−1^)^36^ and *Bacillus cereus* GS-5 (2.7 mg L^−1^ h^−1^)^31^. The formation of N–NO_2_ was detected in DM, which was a result from the oxidation of N–NO_3_. A maximum concentration of N–NO_2_ was observed at 16 h (18.66 ± 1.68 mg L^−1^ h^−1^) and decreased until it was completely utilized in 20 h of incubation (Fig. 2d). However, N–NO_3_ was not completely removed at the end of the assay (13.54 ± 0.60 mg L^−1^ in 32 h; Fig. 2d), suggesting that the denitrification process by Str21 was partially inhibited by the aerobic condition.

#### Ammonium and nitrate transformation in simultaneous nitrifying-denitrifying medium

Bzr02 and Str21 were cultivated in simultaneous nitrification–denitrification medium (SNDM) under aerobic conditions, and their growth and utilization of N–NH_4_ and N–NO_3_ were followed for 36 h (Fig. 2e,f). The average and maximum removal rates of N–NH_4_ and N–NO_3_ for both strains were described in Table 2. Bzr02 was able to remove 94.1 ± 1.3% of N–NH_4_ and 70.2 ± 3.6% of N–NO_3_ after 36 h of incubation (Table 2). A total of 16.80 ± 1.24 mg L^−1^ of N–NO_3_ was accumulated in SNDM after 24 h of incubation, with no further utilization by Bzr02 (Fig. 2e). The formation of N–NO_2_ was detected in SNDM, which was a result from the N–NO_3_ oxidation. The concentration of N–NO_2_ increased to a maximum of 20.02 ± 1.15 mg L^−1^ after 6 h, however, 9.48 ± 0.99 mg L^−1^ of N–NO_2_ remained accumulated in SNDM from 24 h of incubation (Fig. 2e). The average removal rate of N–NH_4_ (5.07 ± 0.09 mg L^−1^) was significantly higher than N–NO_3_ (1.44 ± 0.16 mg L^−1^), which suggests that Bzr02 preferred to utilize N–NH_4_ in SNDM (Table 2).

Similarly, Str21 was able to remove a higher amount of N–NH_4_ (95.6 ± 1.5%) than of N–NO_3_ (75.4 ± 2.6%) (Table 2), however, the utilization of N–NO_3_ was not significant until after 12 h of incubation (Fig. 2f). The formation of N–NO_2_ was detected in SNDM, which was a result from the reduction of N–NO_3_, however, some differences were observed when Str21 was compared to Bzr02: (i) the maximum concentration of N–NO_2_ was lower (12.19 ± 0.77 mg L^−1^) and it was observed after 12 h of incubation, and (ii) N–NO_2_ was almost completely utilized after 24 h of incubation (Fig. 2f). Moreover, a lower concentration of N–NO_3_ (12.07 ± 0.91 mg L^−1^) was accumulated after 24 h of incubation (Fig. 2f), when compared to Bzr02. The average removal rate of N–NH_4_ (3.35 ± 0.04 mg L^−1^) was higher than of N–NO_3_ (2.29 ± 0.22 mg L^−1^), which also suggested that Str21 preferred to utilize N–NH_4_ in SNDM (Table 2). Similar results for other strains, where the removal rate of N–NH_4_ was faster than of N–NO_3_, have been described for *Klebsiella pneumoniae* CF-S9 (3.3 and 2.6 mg L^−1^, respectively)^36^ and *Pseudomonas tolaasii* Y-11 (2.13 and 0.52 mg L^−1^, respectively)^39^. However, other strains have been found to remove N–NO_3_ faster than of N–NH_4_, i.e.: *Bacillus cereus* GS-5 (2.94 and 2.69 mg L^−1^, respectively)^31^ and *Janthinobacterium svalbardensis* F19 (1.19 and 0.62 mg L^−1^, respectively)^20^.

#### Hydroxylamine influence in the ammonium transformation by the strain Bzr02 in nitrifying medium

**Figure 3:**
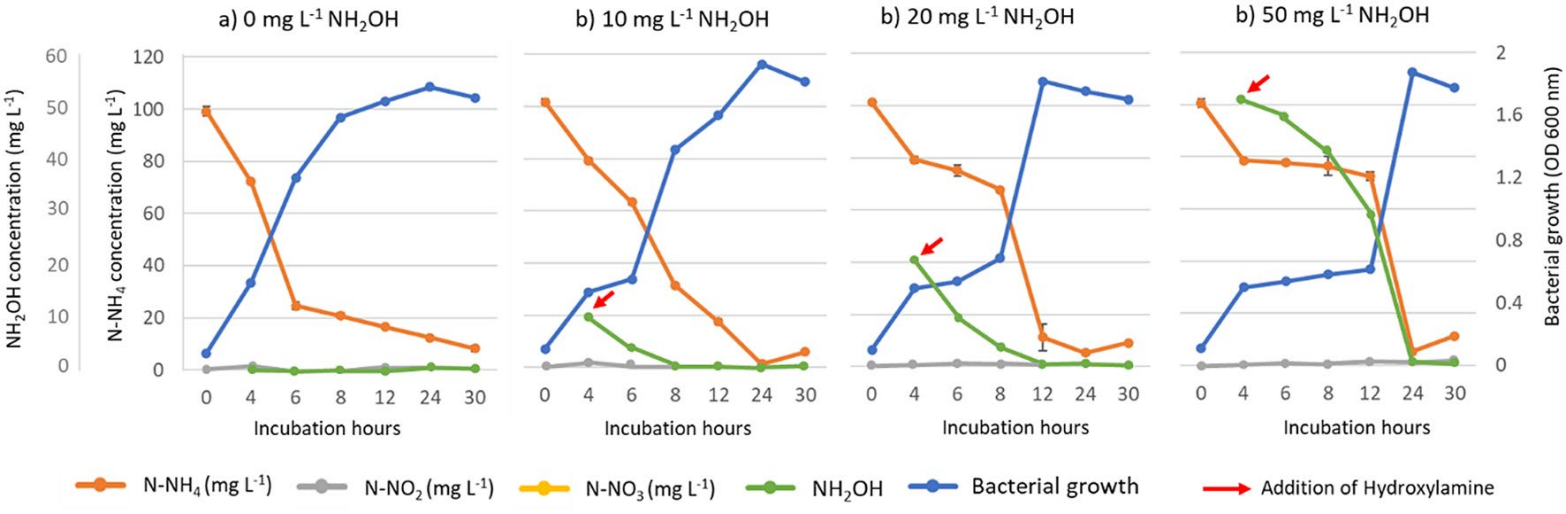
Dynamics of ammonium transformation with addition of hydroxylamine in different concentrations—strain Bzr02, (**a**) 0 mg L^-1^ or control, (**b**) 10 mg L^−1^, (**c**) 20 mg L^−1^ and (**d**) 50 mg L^−1^. Values represent the mean and the standard error (n = 3).

Bzr02 was cultivated in NM supplemented with NH_2_OH in different concentrations, and the growth and utilization of N–NH_4_ and NH_2_OH were followed for 30 h (Fig. 3). The experiment was performed to corroborate the nitrification process by Bzr02 since the oxidized products (N–NO_2_ and N–NO_3_) were not observed during incubation with N–NH_4_ as the sole nitrogen source. Bzr02 presented a log phase after 4 h of incubation in the control medium without hydroxylamine, which also corresponded with the maximum removal rate of N–NH_4_ (23.80 ± 0.84 mg L^−1^, Fig. 3a). When NH_2_OH was added to 10 mg L^−1^ in NM after 4 h of incubation, the log phase of Bzr02 was observed until after 6 h of incubation (Fig. 3b). The maximum removal of N–NH_4_ was 8.03 ± 0.60 mg L^−1^ h^−1^ during the addition of 10 mg L^−1^ NH_2_OH, which was significantly lower when compared to the control (Fig. 3a,b). When 20 and 50 mg L^−1^ of NH_2_OH were added to NM after 4 h of incubation, the log phase was observed after 8 and 12 h of incubation, respectively (Fig. 3c,d). Moreover, the maximum removal rates of N–NH_4_ were 2.05 ± 0.90 and 0.86 ± 0.67 mg L^−1^, respectively, which were significantly lower when compared to the control (Fig. 3a,c,d). These results suggested that NH_2_OH, in high concentrations, significantly inhibited the growth of Bzr02, and in consequence, the removal of N–NH_4_. However, the transformation of N–NH_4_ was resumed when significant amount of NH_2_OH was removed by Bzr02. Furthermore, N–NO_2_ was not detected as product from the oxidation of NH_2_OH (Fig. 3b,c,d). Similar results in other strains have been reported for: *Enterobacter cloacae* CF-S27^22^, *Alcaligenes faecalis*^21^, and *Thiosphaera pantotropha* (formerly *Paracoccus denitrificans*)^42^.

#### Confirmation of bacterial nitrogen transforming pathways

**Figure 4:**
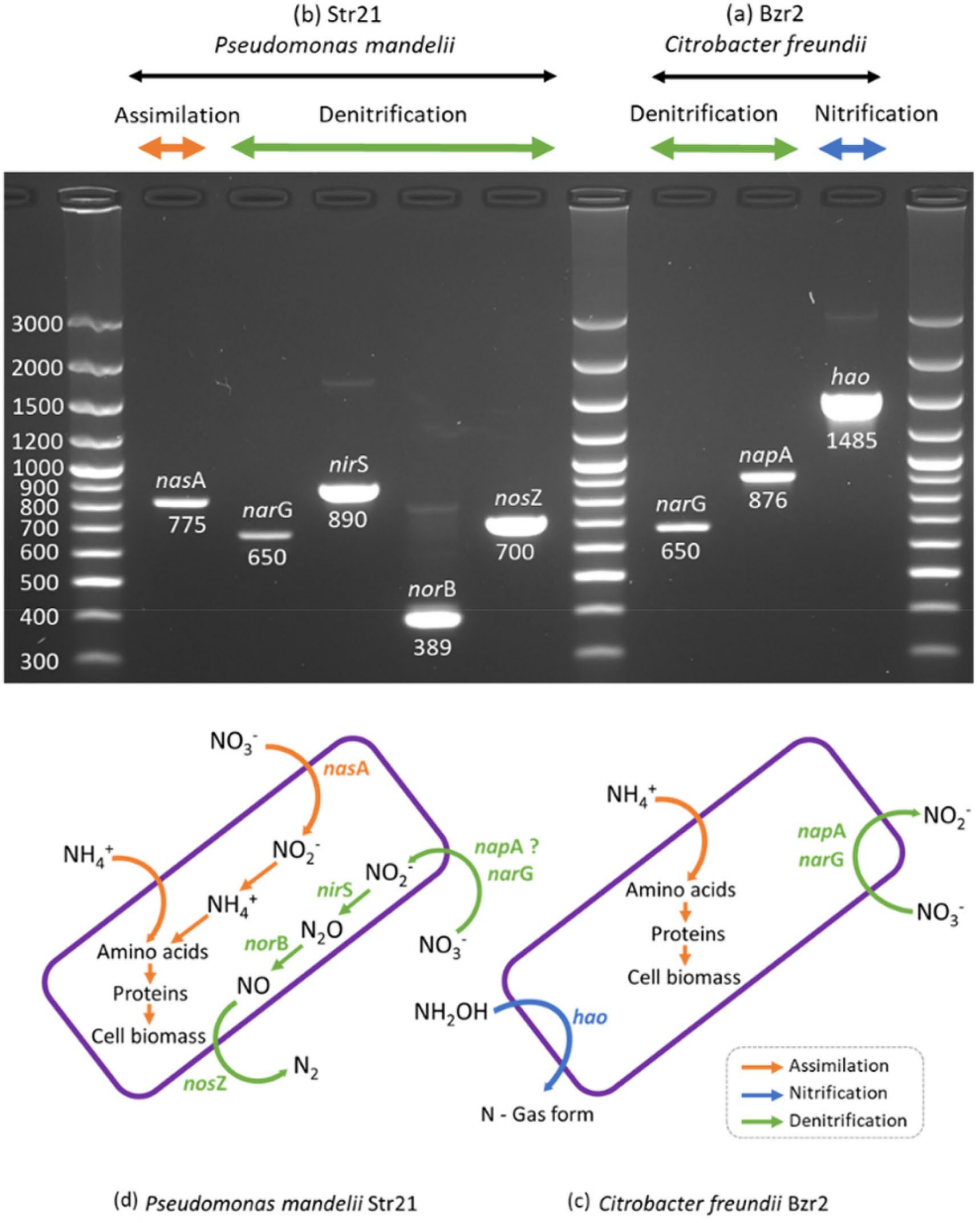
PCR amplification of key functional genes involved in nitrogen transformations for bacterial strains (**a**) Str21 and (**b**) Bzr02, and the predicted nitrogen utilization pathways in (**c**) Str21 and (**d**) Bzr02.

The nitrogen balance during the transformation processes for Bzr02 and Str21 was calculated and presented in Table 3. The detection of key functional genes involved in nitrogen cycling was also summarized in Fig. 4, and the results were used to corroborate their nitrogen transforming pathways. For the ammonium transformation assay using NM, Bzr02 and Str21 utilized almost complete nitrogen and incorporated it into their cell biomass (94.7 ± 1.4 and 94.3 ± 2.0 mg L^−1^, respectively) (Table 3). Only a small fraction of nitrogen was lost for Bzr02 and Str21 (0.75 and 1.25 mg L^−1^, respectively; Table 3), suggesting that it was assimilated when N–NH_4_ was given as the sole nitrogen source. The nitrification process seemed not to have occurred, especially because the products from the oxidation of N–NH_4_ (N–NO_2_ and N–NO_3_) were not significantly detected through the entire assays (Fig. 2a,b).

**Table 3:** Nitrogen balance of strains Bzr02 and Str21 during the nitrogen transformation.

Media	Strain	Initial TN (mg L^−1^)	Final TN (mg L^−1^)		Lost N (mg L^−1^)
			Extracellular	Intracellular	
NM	Bzr02	98.4 ± 0.6	2.55 ± 0.85	94.7 ± 1.4	0.75
	Str21	98.0 ± 0.9	2.45 ± 1.06	94.3 ± 2.0	1.25
DM	Bzr02	101.9 ± 2.1	98.3 ± 1.4	1.4 ± 0.55	2.2
	Str21	105.0 ± 1.2	11.40 ± 0.62	68.2 ± 1.2	25.4
SNDM	Bzr02	98.9 ± 1.3	29.2 ± 2.1	68.3 ± 1.8	1.4
	Str21	101.5 ± 1.6	14.6 ± 0.2	74.3 ± 1.6	12.6

NM: nitrifying medium; DM: denitrifying medium; SNDM: simultaneous nitrifying-denitrifying medium. Values represent the mean and the standard error (n = 3).

The nitrification process seemed to have occurred for Bzr02 when NH_2_OH was added to NM, which is another intermediary product during the first reaction of nitrification (NH_4_^+^  → [NH_2_OH] → NO_2_^−^). Bzr02 removed NH_2_OH from the NM while there was no significant bacterial growth or removal of N–NH_4_ (Fig. 3), suggesting that NH_2_OH was oxidized (nitrification) rather than assimilated. Additionally, the detection of the gene *hao* (hydroxylamine oxidoreductase, HAO) supports the nitrification process by Bzr02 (Fig. 4a); however, the concentration of N–NO_2_ -the product from NH_2_OH oxidation- was not significantly detected in all experiments (Fig. 3). These results are different from other strains that produced NO_2_^−^ from the oxidation of NH_2_OH, i.e., *Nitrosomonas europaea*^18^ and *Pseudomonas* PB16^23^. Other studies suggest that the enzyme HAO also catalyzes a different reaction where NH_2_OH is transformed to nitric oxide (NO) in *Alcaligenes faecalis* No.4^43^ or reduced to N_2_ in *A. facecalis*^44,45^ and *Acinetobacter calcoaceticus* HNR^19^. The above results suggests that Bzr02 could have reduced NH_2_OH to a nitrogen gas (Fig. 4b), rather than being oxidized to NO_2_^−^ in the process of nitrification.

In the nitrate transformation assay in DM, only Str21 was able to grow and utilize N–NO_3_ as the only nitrogen source (Fig. 2d). The initial nitrogen content in DM (105.0 ± 1.2 mg L^−1^) was utilized by Str21 until 11.40 ± 0.62 mg L^−1^ remained in the medium at the end of the experiment (Table 3). The majority of nitrogen was detected in the cell biomass of Str21 (68.2 ± 1.2 mg L^−1^) and 25.4 mg L^−1^ was estimated to be lost (Table 3). The above results suggested that Str21 transformed 89.1% of total nitrogen, from which 65.0% was assimilated and the remaining 24.1% was probably lost as a nitrogen gaseous form in the process of denitrification. Str21 was found to contain the gene *nas*A (assimilatory nitrate reductase, NAS; Fig. 4b) that confirmed the process of assimilatory NO_3_^−^ reduction to NO_2_^−^, and subsequently to NH_4_^+^. The gene *nas*A is involved in the synthesis of cell biomass^46^ (Fig. 4d). Moreover, Str21 was found to contain all studied genes involved in the process of denitrification (*nar*G, *nir*S, *nor*B and *nos*Z; Fig. 4b), suggesting that it is a facultative anaerobic denitrifier (Fig. 4d). The denitrification activity in anaerobic conditions for a similar strain—*Pseudomonas mandelii* strain PD30—has already been described with the gene expression of *nir*S and *nor*B^47,48^. In the above research, it was argued that the gene expression was significantly inhibited in aerobic conditions, and therefore, it was concluded that *P. mandelii* PD30 performed denitrification in exclusive anaerobic conditions. In contrast, for other *Pseudomonas* strains, i.e., *P. stutzeri* YG-24^29^, *P.* sp. JQ-H3^33^ and *P. mendocina* GL6^49^, the removal of nitrogen content as gas was up to 46.0—74.4% in aerobic conditions, suggesting that there was a similar preference for nitrogen denitrification and assimilation, and sometimes, denitrification could be significantly higher. The detection of the gene *nap*A, rather than the gene *nar*G, was probably the most important factor influencing aerobic denitrification in the above three mentioned strains. In the case of Str21, only the gene *nar*G was detected (Fig. 4b), however, the process of denitrification was not completely inhibited when it was incubated in DM, during aerobic conditions (Fig. 2d). We believe that the aerobic conditions in the media could have partially influenced the reduction of N–NO_3_ to subsequent forms of nitrogen for Str21, resulting in an evident preference to assimilate nitrogen rather than performing denitrification.

For the N–NH_4_ and N–NO_3_ transformation assays in SNDM, Bzr02 and Str21 were able to utilize N–NH_4_ and N–NO_3_ in aerobic conditions. For Bzr02, a total of 68.3 ± 1.2 mg L^−1^ of nitrogen was found in the cell biomass and 29.2 ± 2.1 mg L^−1^ remained in the medium (Table 3). The remaining nitrogen was mostly from N–NO_3_ and the accumulation of its reduction to N–NO_2_, that were not completely depleted by Bzr02 (Fig. 2e). The above results could be associated from the difficulty of Bzr02 to reduce NO_3_^−^ to NO_2_^−^ in aerobic conditions, as it was explained when it was incubated with higher N–NO_3_ concentrations in DM (Fig. 2c). Despite the above, only 1.4 mg L^−1^ of nitrogen was lost (Table 3), suggesting that the dominant metabolic pathway presented by Bzr02 was nitrogen assimilation (Fig. 4c). The gene *nas*A was not detected for Bzr02, indicating that N–NO_3_ was rather reduced by a dissimilatory nitrate reductase (NAR or NAP), and then, part of N–NO_2_ was incorporated into the cell biomass through the process of assimilatory nitrite reduction^46,50^ (Fig. 4c).

Str21 presented 74.3 ± 1.6 mg L^−1^ of nitrogen in the cell biomass and 14.6 ± 0.2 mg L^−1^ remained in the medium with no further utilization (Table 3). A significant concentration of nitrogen (12.6 mg L^−1^) was lost at the end of incubation for Str21 (Table 3) in comparisson to Bzr02, suggesting that the process of denitrification took place. Moreover, the N–NO_2_—produced from the reduction of N–NO_3_—was not accumulated in Strs21 as it was observed for Bzr02 (Fig. 2e,f), also supporting that N–NO_2_ was further reduced into nitrogen gaseous forms in the process of denitrification. Similarly as it was described during the experiment in DM, the detection of *nas*A suggested that N–NO_3_ was incorporated into the cell biomass through the process of assimilatory nitrate reduction, and the detection of all four nitrogen reductase genes (*nar*G, *nir*S, *nor*B and *nos*Z) supported that the lost nitrogen escaped as nitrogen gas during dissimilatory nitrate reduction (denitrification; Fig. 4d). The low denitrification activity by Str21 in SNDM was also the influece of the aerobic conditions, which could be appreciated for the long lag phase were N–NO_3_ was not significantly utilized at the first 12 h of incubation (Fig. 2d).

## Conclusion

Bzr02 and Str21 (isolated from SSBSs sediments), identified as *Citrobacter freundii* and *Pseudomonas mandelii*, respectively, were found to have potential applications in nature-based solutions to enhance nitrogen compounds removal, such as SSBSs. Nitrate reduction to nitrite in the denitrification process was found for both strains. Str21 seemed to be a facultative anaerobic denitrifier, and therefore, could participate in nitrogen cycling in SSBSs sediments, where oxygen limiting conditions occur. In turn, Bzr02 and Str21 were observed to significantly assimilate N–NH_4_ and N–NO_3_ into their cell biomass in aerobic conditions, which could subsequently help to improve the efficiency of SSBSs in the nitrogen removal with its sequestration in the sediments. Therefore the application of both strains could be recommended for sedimentation zones, where the release of nitrogen would be controlled by: i) other decomposing microbial communities dwelling in the sediments, and ii) the periodical removal of sediments to maintain the proper operation of SSBSs.

## Materials and methods

### Samples collection and isolation of bacteria

Sediment samples were collected from the sedimentation zone (August 2018) in three SSBSs constructed for different urban rivers: (i) the River Sokołówka (Sok-SSBS) and (ii) the River Bzura (Bzr-SSBS) in the city of Łódź, and (iii) the River Struga Gnieźnieńska (Str-SSBS) in the city of Gniezno, Poland.^9^ Complete description of structure and function for Bzr-SSBS is detailed in Szulc et al.^51^ and Jurczak et al.^8^, and for Sok-SSBS and Str-SSBS in Font-Nájera et al.^9^ Sediment samples were suspended in sterile 0.75% NaCl w/v (10 g of sediment in 90 mL) and shacked for 30 min at 25 °C. Samples were allowed to settle for 15 min and supernatant was used to prepare serial dilutions (1 × 10^–1^–1 × 10^–6^) according to Mankiewicz-Boczek et al.^52^. 100 µL of each dilution was plated on to Soil Extract Agar (SEA), a solid medium according to Hamaki et al.^53^, and incubated for seven days at 25 °C. For each SSBS, 50 heterotrophic bacterial isolates (150 in total) were randomly streaked out and re-plated on to nutrient agar solid medium (NA, Karl Roth).

### Screening of nitrogen transforming bacteria

A total of 150 well-separated bacterial colonies were picked from NA and checked for nitrogen transformation abilities in different culturable media (See also media description in supplementary material):

(i) in Giltay denitrifying medium (GiDM) with high content of NO_3_^−^ (N: 277 mg L^−1^) according to Alexander^54^, at 25 °C. Bacterial ability to reduce NO_3_^−^, under oxygen limited condition (Becton Dickinson Gas Pak System), was qualitatively monitored every 12 h with the semi-quantitative test strips QUANTOFIX nitrate/nitrite (Macherey–Nagel) for 7 d. A total of 10 different bacterial strains were able to completely or partially reduce NO_3_^−^ (denitrification process), and therefore, were selected for further experiments;

(ii) the 10 selected bacterial isolates were incubated in 15 mL glucose nitrifying medium (GNM) described in Pahdi et al.^22^, with a small modification—KH_2_PO4 was used instead of NaH_2_PO4 (0.10 g MgSO_4_ · 7H_2_O, 3.84 g K_2_HPO_4_, 1.5 g KH_2_PO_4,_ 0.802 g NH_4_Cl [N: 212 mg L^−1^], 5.3 g glucose C_6_H_12_O_6_ [C: 2120 mg L^−1^]), and 2 mL of trace elements were added per 1000 mL of GNM, final pH was 7.2, shacked at 150 rpm and incubated at 25 °C. The trace element solution was prepared according to Pahdi et al.^22^. The effect of different carbon sources was also screened with changes to the nitrifying medium where glucose was replace by: (i) sodium succinate (11.9 g)—succinate nitrifying medium (SNM), (ii) sodium acetate (10.0 g) – acetate nitrifying medium (ANM), and (iii) sodium citrate (8.65 g)—citrate nitrifying medium (CNM). The carbon and nitrogen ratio was kept constant (C:N = 10) in all used media.

Bacteria were also tested for the transformation of NH_4_^+^ under the presence of hydroxylamine in GNM. Cultures were grown in GNM for 6 h and spiked with high concentration of hydroxylamine (100 mg L^−1^ final concentration) according to Padhi et al.^22^. For the screening purpose, their ability to transform NH_4_^+^ was qualitatively monitored with the semi-quantitative test strips QUANTOFIX ammonium (Macherey–Nagel), every 12 h during 7 d.

### DNA isolation and detection of key functional genes involved in nitrogen transformation processes

DNA was isolated from overnight bacterial cultures (Luria Bertani broth, LB) according to the specification in Wizard Genomic DNA purification kit (Promega, Madison, Wisconsin). The 10 previously selected bacterial strains (Chapter 2.2.) were screened for the presence of key functional genes involved in nitrification (*hao*^22^), denitrification (*nap*A^38^, *nar*G^55^, *nir*S^56^, *nor*B^57^ and *nos*Z^58^) and nitrogen assimilation (*nas*A^59^) processes using conventional PCR (Supplementary Table S4). PCR products for the strains Bzr02 and Str21 were purified with the QIAEX II Gel Extraction Kit (Promega, Madison, Wisconsin) and sequenced by Genomed laboratories in Warsaw, Poland (http://www.genomed.pl/). DNA sequences were edited using the software MEGA7 (http://www.megasoftware.net/) and similarity with other published bacterial strains was verified with the nucleotide BLAST tool. Sequences were deposited in the GenBank database with the accession numbers for Str21: *nos*Z (MW286255), *cnor*B (MW286256), *nir*S (MW286257), *nar*G (MW286258), and *nas*A (MW286259), and for Bzr02: *nap*A (MW286261), *hao* (MW286262), and *nar*G (MW286263).

### Taxonomic characteristics and phylogenetic analysis

The 16S rRNA bacterial molecular marker was amplified for the 10 selected strains, with 27F / 1492R primers according to Lane^60^. PCR products were processed (purification, sequencing and nucleotide BLAST analysis) similarly as specified for the functional genes in Chapter 2.3. A neighbour-joining phylogenetic tree was constructed for bacteria using the software MEGA7. Bacterial 16S rRNA sequences were deposited in the GenBank database with the accession numbers for Str21 (MW282158), Bzr02 (MW282159), Bzr07 (MW282160), Str01 (MW282161), Sok03 (MW282162), Sok05 (MW282163), Sok41 (MW282164), Sok01 (MW282165), Sok06 (MW282166), and Sok20 (MW282167).

Additional methods to corroborate the taxonomical identification of two strains (Bzr02 and Str21) were described in supplementary material. The strain Bzr02 was incubated on GEN III Biolog MicroPlates with different carbon substrates, according to the manufacturer specifications^61^, and the taxonomic characteristics of bacterium were determined using the GEN III Biolog database. For Str21, the gene *rpo*B (coding for the β sub-unit of the RNA bacterial polymerase) was used as a molecular marker, since it has been recommended for the optimal differentiation between *Pseudomonas* species^62^. The DNA sequence of the *rpo*B gene was published in GenBank database for Str21 (MW286260).

### Ammonium transformation

Bzr02 and Str21 were cultured overnight in LB at 25 °C and 120 rpm. Cells were harvested by centrifugation (8000 rpm, 10 min, 4 °C), and washed three times with sterile water. Then, each strain was inoculated into the nitrifying medium NM (0.1 final OD_600_) with adjusted concentrations of NH_4_^+^ (N: 100 mg L^−1^) and glucose (C: 1000 mg L^−1^), incubation was performed at 25 °C and 150 rpm. Bacterial growth (optical density OD 600 nm) was checked at 2 h intervals using an Eppendorf Biophotometer in a 24 h experiment^22^. Supernatant was also collected during each interval (13,000 rpm, 10 min, 4 °C) for the measurement of N–NH_4_, N–NO_3_, N–NO_2_ and extracellular TN. The pellet was washed three times with sterile water and used to estimate intracellular TN^32^.

### Nitrate transformation

Bzr02 and Str21 were inoculated into denitrifying medium (DM). The denitrifying media was similar to NM with the use of KNO_3_ (N: 100 mg L^−1^ final concentration) as the source of nitrogen. The check of bacterial growth and the collection of samples were performed similarly as explained for the ammonium transformation assays in a 32 h experiment^22^. The supernatant was used to measure N–NH_4_, N–NO_3_, N–NO_2_ and extracellular TN, and the bacterial pellet for intracellular TN.

### Simultaneous ammonium and nitrate transformation

Bzr02 and Str21 were inoculated into the simultaneous nitrifying-denitrifying medium (SNDM). The media was similar to NM with the use of KNO_3_ and NH_4_CL (N: 50 mg L^−1^ each; TN: 100 mg L^−1^ final concentration) as sources of nitrogen. The check of bacterial growth and the collection of samples was performed similarly as explained for the ammonium transformation assay, in a 50 h experiment^22^. The supernatant was used to measure N–NH_4_, N–NO_3_, N–NO_2_ and extracellular TN, and the bacterial pellet for intracellular TN.

### The impact of hydroxylamine for ammonium transformation

Bzr02 was the only strain capable of growth in the presence of hydroxylamine during the screening experiments (described in Chapter 2.2.). Therefore, in a parallel experiment, the transformation of ammonium by Bzr02 was also investigated with different concentrations of hydroxylamine (0, 10, 20 and 50 mg L^−1^ as final concentrations) added after 4 h of growth in NM. The bacterial growth and the collection of samples were performed similarly as explained for the ammonium transformation assay, at 0 and 2 h (before the addition of hydroxylamine), and 4, 8, 12, 24 and 30 h of incubation (after the addition of hydroxylamine)^22^. The supernatant was used to measure N–NH_4_, N–NO_3_, N–NO_2_ and NH_2_OH concentrations.

### Analytical methods

Concentration of nitrogen sources were measured with the Multiskan Sky Microplate Spectrophotometer (Thermo Fisher Scientific) according to standard methods^63^: (i) N–NH_4_ by the Nessler’s colorimetric assay, (ii) N–NO_3_ by the ultraviolet spectrophotometric method, and (iii) N–NO_2_ by the Griess colorimetric assay. The Hydroxylamine was measured by indirect spectrophotometry^64^. The TN was calculated with the total Kjeldal reagent set^65^ as follows: (i) using the supernatant for the extracellular TN, and (ii) reconstitution of the cell pellet with sterile water for intracellular TN^32^. All measurements were performed in triplicate.

### Analysis of data

Nitrogen balance was monitored with the formula:

$$ {\text{N}}_{{\text{L}}} = \left( {{\text{TN}}_{{{\text{Fe}}}} + {\text{ TN}}_{{{\text{Fi}}}} } \right){-}{\text{TN}}_{{{\text{Ie}}}} $$

where *N*_*L*_ is the loss of nitrogen at the end of the experiment, the *TN*_*Fe*_ and *TN*_*Fi*_ are the final extracellular and intracellular TN, respectively, and the *TN*_*Ie*_ is the initial extracellular TN (adapted from Fidélis Silva et al.^32^).

Bacterial removal rates for N–NH_4_^−^, N–NO_3_^−^ and NH_2_OH (mg L^−1^ h^−1^) were estimated as follows:

$$ \begin{aligned}  &{{\text{Rr}}\left( {{\text{mg}}\;{\text{L}}^{ - 1} \;{\text{h}}^{ - 1} } \right) = \, \left( {{\text{C}}_{{\text{i}}} {-}{\text{ C}}_{{\text{f}}} } \right)/{\text{t}},\;{\text{and}}} \hfill \\&\quad {{\text{Rr}}\left( \% \right) \, = \, 100 \times \left( {{\text{C}}_{{\text{i}}} {-}{\text{ C}}_{{\text{f}}} } \right)/{\text{C}}_{{\text{i}}} }  \end{aligned} $$

where *C*_*i*_ and *C*_*f*_ are the initial and final concentration of the nitrogen source, respectively, and the *t* is the final time of the experiment^29^.

## Supplementary Information

Supplementary Information
