# Supplementary material for: Culturable nitrogen-transforming bacteria from sequential sedimentation biofiltration systems and their potential for nutrient removal in urban polluted rivers
Source: Sci Rep. 2021 Apr 2;11:7448. doi: 10.1038/s41598-021-86212-3 (PMC8018948; doi:10.1038/s41598-021-86212-3)
Supplement: Supplementary file 1 — Supplementary Information [file 41598_2021_86212_MOESM1_ESM.docx]

**SUPPLEMENTARY MATERIAL**

**Culturable nitrogen-transforming bacteria from sequential sedimentation biofiltration systems and their potential for nutrient removal in urban polluted rivers**

Font Nájera Arnoldo^a,b,*^, Serwecińska Liliana^b^, Mankiewicz-Boczek Joanna^a,b^

^a^ University of Lodz, Faculty of Biology and Environmental Protection, UNESCO Chair on Ecohydrology and Applied Ecology, Banacha 12/16, 90-237 Łódź, Poland.

^b^ European Regional Centre for Ecohydrology of the Polish Academy of Sciences, Tylna 3, 90-364 Łódź, Poland.

***Corresponding author:** Serwecińska Liliana

e-mail: l.serwecinska@erce.unesco.lodz.pl

Arnoldo Font Nájera [a.font-najera@erce.unesco.lodz.pl](mailto:a.font-najera@erce.unesco.lodz.pl)

Joanna Mankiewicz-Boczek [j.mankiewicz@erce.unesco.lodz.pl](mailto:j.mankiewicz@erce.unesco.lodz.pl)

**Table S1**. Nucleotide BLAST sequence identity analysis for the gene 16S rRNA of nitrogen transforming bacterial strains.

| **SSBS** | **#** | **Strain** | **Query cover (%)** | **Identity (%)** | **Result** | **Accession** |
| --- | --- | --- | --- | --- | --- | --- |
| Sok | 1 | Sok01 | 100 | 99.32 | *Hydrogenophaga taeniospiralis* NBRC 102512 | AB681846 |
|  | 2 | Sok03 | 98 | 99.45 | *Pseudomonas guineae* LMGT 24016 | AM491810 |
|  | 3 | Sok06 | 100 | 99.63 | *Bacillus aerius* 24K | AJ831843 |
|  | 4 | Sok20 | 99 | 99.34 | *Janthinobacterium lividum* DSM 1522 | NR_164625 |
|  | 5 | Sok41 | 99 | 99.29 | *Acidovorax radicis* N35 | NR_117776 |
|  | 6 | Sok05 | 100 | 99.08 | *Kocuria rosea* DSM 20447 | NR_044871 |
| Bzr | 7 | **Bzr02** | 99 | 99.39 | ***Citrobacter freundii* NBRC 12681** | NR_113596 |
|  | 8 | Bzr07 | 100 | 99.83 | *Pseudomonas migulae* NBRC 103157 | NR_114223 |
| Str | 9 | Str01 | 100 | 98.36 | *Bacillus simplex* LMG 11160 | NR_114919 |
|  | 10 | **Str21** | 99 | 99.55 | ***Pseudomonas mandelii* NBRC 103147** | NR_114216 |

Sok: Sokolówka SSBS, Bzr: Bzura-SSBS, Str: Struga Gnieznienska SSBS.

**
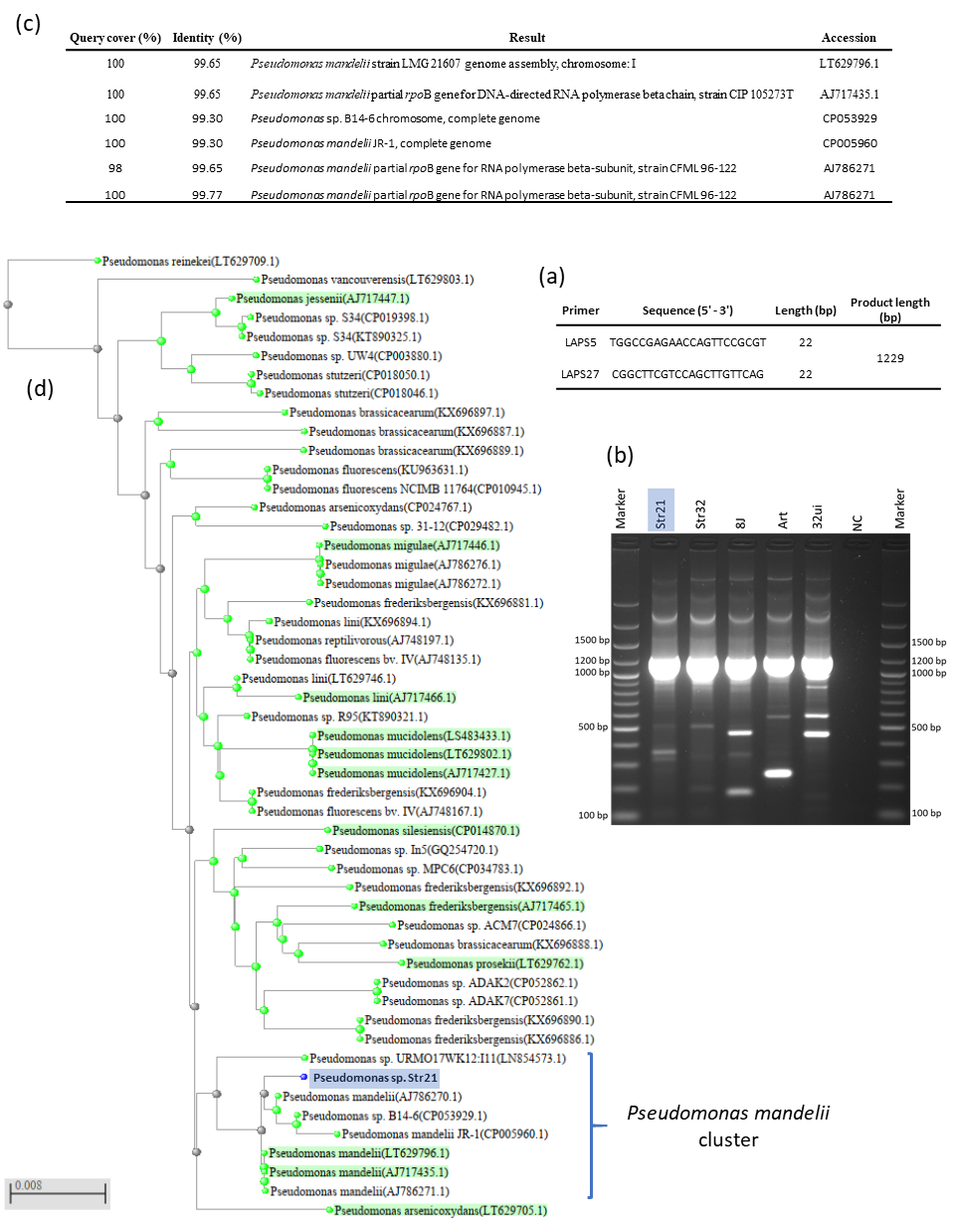
**

**Fig S1.** Taxonomical identification of strain Str21 with the detection and sequence analysis of gene encoding for the β subunit of bacterial RNA polymerase (*rpo*B); (a) description of primer sets, (b) detection in agarose gel electrophoresis using conventional PCR, (c) nucleotide BLAST similarity results, and (d) Phylogenetic analysis with other related *Pseudomonas* spp. published strains. PCR conditions for primer sets were described in Tayeb et al., (2005). Molecular marker Blirt-Gdansk (100-3000 bp); positive *Pseudomonas* spp. strains: Str32, 8J, Art, 32UI. NC: negative control. Strains highlighted in green were retrieved from full genomic published sequences. The strain Str21, highlighted in blue, is closely associated to other strains that have been identified as *Pseudomonas mandelii*, and therefore, the bacterium Str21 was believed to be a different strain of the same species.

**Table S2.** Taxonomical identification of strain Bzr02 according to the metabolic profile obtained in BIOLOG GEN III MicroPlates.

|  | **Protocol A** | | | |  |  | **Protocol B** | | | |
| --- | --- | --- | --- | --- | --- | --- | --- | --- | --- | --- |
| No. | PROB | SIM | DIST | Best results |  | No. | PROB | SIM | DIST | Best results |
| **24h of incubation** | | | | |  |  | **24h of incubation** | | | |
| 1 | 0.803 | 0.803 | 2.778 | *Citrobacter freundii* |  | 1 | 0.646 | 0.646 | 5.114 | *Citrobacter freundii* |
| 2 | 0.325 | 0.325 | 2.979 | *Citrobacter braakii* |  | 2 | 0.126 | 0.126 | 5.655 | *Citrobacter sedlakii* |
| 3 | 0.007 | 0.007 | 5.318 | *Citrobacter youngae* |  | 3 | 0.096 | 0.096 | 5.821 | *Citrobacter braakii* |
| 4 | 0.007 | 0.007 | 5.388 | *Citrobacter sedlakii* |  | 4 | 0.091 | 0.091 | 5.846 | *Citrobacter youngae* |
|  |  |  |  |  |  |  |  |  |  |  |
| **48h of incubation** | | | | |  |  | **48h of incubation** | | | |
| 1 | 0.865 | 0.865 | 1.921 | *Citrobacter freundii* |  | 1 | 0.717 | 0.526 | 3.762 | *Citrobacter freundii* |
| 2 | 0.37 | 0.37 | 2.072 | *Citrobacter braakii* |  | 2 | 0.135 | 0.09 | 4.833 | *Citrobacter braakii* |
| 3 | 0.005 | 0.005 | 4.633 | *Citrobacter werkmanii* |  | 3 | 0.127 | 0.084 | 4.873 | *Citrobacter sedlakii* |
| 4 | 0.004 | 0.004 | 4.894 | *Citrobacter youngae* |  | 4 | 0.021 | 0.012 | 6.029 | *Citrobacter youngae* |

The isolated strain Bzr02 was incubated into plates using two different liquid media: protocol A) for general metabolic description of heterotrophic aerobes, and Protocol B) to identify a false positive from the results obtained in protocol A, according to the manufacturer instructions (BIOLOG Hayward, California). The PROB (probability), SIM (similitude) and DIST (distance) indexes were measured at 24h and 48h, and were used to identify the most probable metabolic profile of the strain Bzr02 with the GEN III database provided by BIOLOG. The best results were always observed for the metabolic profile presented by *Citrobacter freundii*, and therefore, the bacterium Bzr02 is believed to be a different strain of the same species.

**Table S3**. Nucleotide BLAST sequence identity analysis for key functional genes detected in strains Bzr02 and Str21.

| **N-transforming process** | **Gene** | **Strain** | **Query cover (%)** | **Identity (%)** | **Result** | **Accession** | **Observations** |
| --- | --- | --- | --- | --- | --- | --- | --- |
|  |  |  |  |  |  |  |  |
| Denitrification | *nos*Z | Str21 | 100 | 99.93 | *Pseudomonas mandelii* JR-1, complete genome, nitrous oxide reductase gene | CP005960 | extracted from full genome |
|  | *nor*B | Str21 | 100 | 94.41 | *Pseudomonas lini* PD 28 *cnor*B gene | DQ420250 |  |
|  | *nir*S | Str21 | 99 | 96.15 | *Pseudomonas lini* PD 28 *nir*S gene | DQ518188 |  |
|  | *nar*G | Bzr02 | 100 | 98.94 | *Citrobacter freundii* complex sp. CFNIH3 chromosome, complete genome, nitrate reductase subunit alpha | CP026235 | extracted from full genome |
|  |  | Str21 | 99 | 98.39 | *Pseudomonas mandelii* strain LMG 21607 genome assembly, chromosome: I, nitrate reductase subunit alpha | LT629796 | extracted from full genome |
|  | *nap*A | Bzr02 | 100 | 95.96 | *Citrobacter freundii* complex sp. CFNIH3 chromosome, complete genome, nitrate reductase catalytic subunit n*ap*A | CP026235 | extracted from full genome |
| Nitrification | *hao* | Bzr02 | 100 | 99.49 | *Citrobacter freundii* complex sp. CFNIH3 chromosome, complete genome, hydroxylamine reductase | CP026235 | extracted from full genome |
| Assimilation | *nas*A | Str21 | 99 | 93.4 | *Pseudomonas* sp. WP5m-11 assimilatory nitrate reductase (*nas*A) gene, partial cds | JX533707 |  |
|  |  |  |  |  |  |  |  |


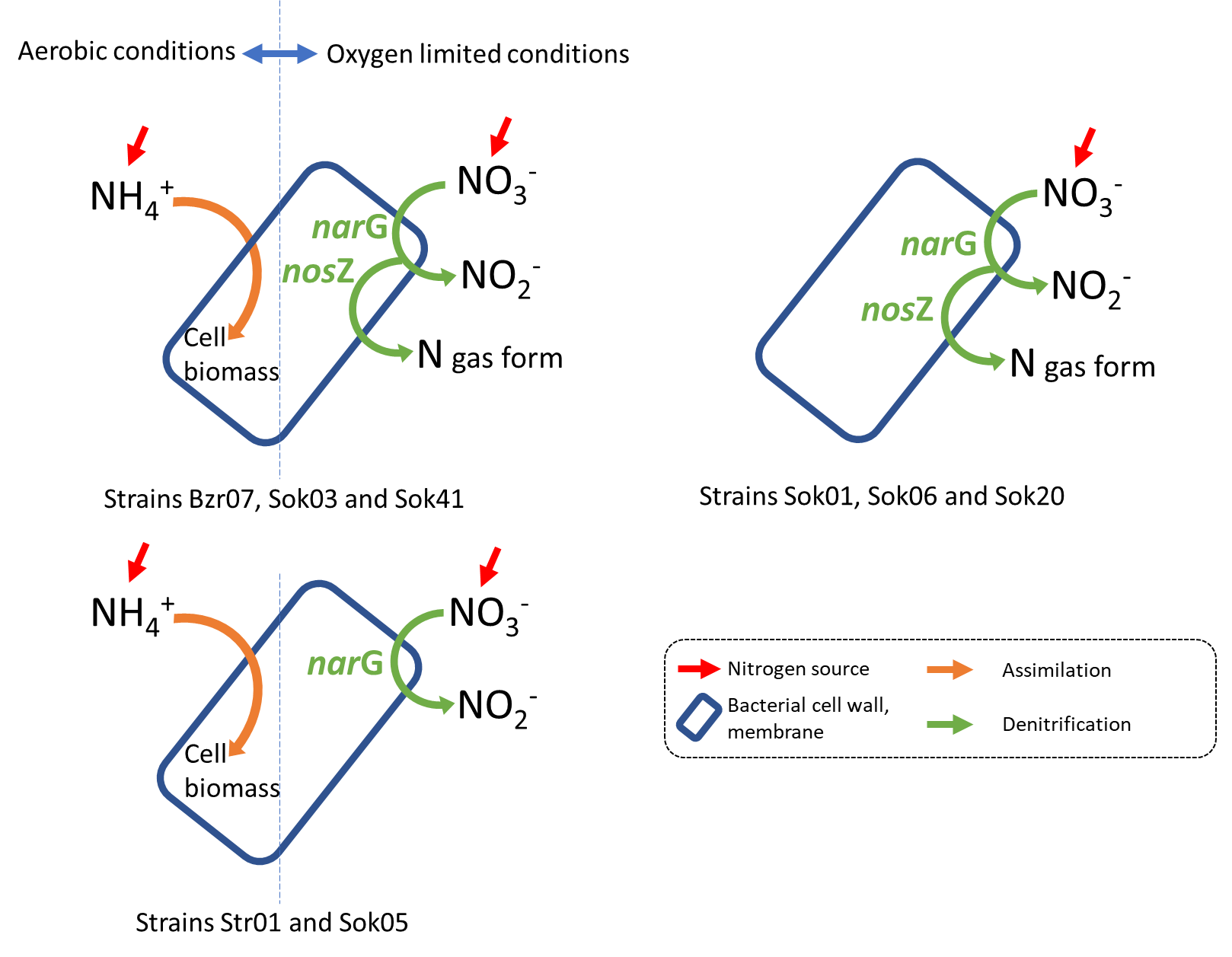


**Fig S2. Metabolic pathways for the transformation of nitrogen compounds in selected bacteria.**

**Supplementary description of media**

1. **Giltay denitrifying medium (GidM), according to Alexander 1965**

Prepare solution 1 and solution 2 separately, in distilled water,

Solution 1 500 mL Solution 2 500 mL

C_4_H_8_N_2_O_3_ 0.05 g Na₃C₆H₅O₇ 2.50 g

Asparagine Trisodium citrate

C_6_H_12_O_6_ 10.0 g KH_2_PO_4_ 2.0 g

Glucose Potassium dihydrogen phosphate

KNO_3_ 2.0g MgSO_4_ 0.97 g

Potassium nitrate Magnesium sulphate

CaCl_2_ 0.1014 g

Mix both solutions and filter sterilize (0.2 µm).

Before inoculation of bacteria, add 45 µL of a stock solution of FeCl (200 mg mL^-1^) for every 50 mL of GidM.

1. **Glucose nitrifying medium (GNM), adapted from Padhi et al., 2017**

Prepare basal and trace elements solution separately,

Basal solution 1000 mL Trace elements solution 1000 mL

MgSO_4_ * 7H_2_O 0.10 g EDTA 1.25 g

Magnesium sulphate heptahydrate

K_2_HPO_4_ 3.84 g ZnSO_4_ 75 mg

Dipotassium phosphate Zink sulphate

KH_2_PO_4_ 1.50 g MnCl_2_ * 4H_2_O 125 mg

Potassium dihydrogen phosphate Manganese (II) chloride dihydrate

NH_4_Cl 0.802 g FeSO_4_ * 7H_2_O 125 mg

Ammonium chloride Iron (II) sulphate heptahydrate

NH_4_Cl 0.802 g CuSO_4_ * 5H_2_O 50 mg

Ammonium chloride Copper (II) sulphate pentahydrate

C_6_H_12_O_6_ 5.3 g CoCl_2_ * 6H_2_O 75 mg

Glucose Cobalt (II) chloride hexahydrate

Adjust the pH to 7.2 and filter sterilize (0.2 µm), for each solution separately.

To prepare working solution (GNM), add 2 mL of trace element solution for every litre of basal medium.

1. **Succinate nitrifying medium (SNM)**

Replace Glucose from the basal medium of GNM with:

11.9 g of C_4_H_4_Na_2_O_4_ * 6H_2_O (sodium succinate dibasic hexahydrate)

1. **Acetate nitrifying medium (ANM)**

Replace Glucose from the basal medium of GNM with:

10.0 g of C_2_H_3_NaO_2_ (sodium acetate)

1. **Citrate nitrifying medium (CNM)**

Replace Glucose from the basal medium of GNM with:

8.65 g of C_6_H_5_Na_3_O_7_ * 2H_2_O (trisodium citrate dihydrate)

1. **Nitrifying medium (NM)**

Modify the content of Glucose and NH_4_Cl from the basal medium of GNM with:

0.3818 g of NH_4_Cl (100 mg L^-1^ of nitrogen content)

2.5000 g of Glucose (1000 mg L^-1^ of carbon content)

1. **Denitrifying medium (DM)**

Modify the content of Glucose and replace NH_4_Cl for NaNO_3_ from the basal medium of GNM with:

0.6068 g of NaNO_3_ (100 mg L^-1^ of nitrogen content)

2.5000 g of Glucose (1000 mg L^-1^ of carbon content)

1. **Denitrifying medium (DM)**

Modify the content of Glucose and NH_4_Cl and add NaNO_3_ from the basal medium of GNM with:

0.1909 g of NH_4_Cl

0.3034 g of NaNO_3_

2.5000 g of Glucose (1000 mg L^-1^ of carbon content)

**Table S4.** PCR primers for detection of bacterial key functional genes.

| **Process** | **Gene** | **Name / Function** | **Primer pair sequence (5’→3’)** | **Size (bp)** | **Thermal cycling conditions** |
| --- | --- | --- | --- | --- | --- |
| Nitrification | *hao* | Hydroxylamine reductase  NH_2_OH → NO_2_  NH_2_OH → N_2_O | Hyrxl_f2  ATGTTTTGTGTNCAATGTGA | 1485^a^ | Initial denaturation at 94°C-5min, followed by 36 cycles of denaturation at 94°C-1min, annealing at 51.8°C-1min, extension at 72°C-1min, and final extension at 72°C-9min. |
|  |  |  | Hyrxl_r2  GCYTTCAGYTCRAACCA |  |  |
| Denitrification | *nap*A | Periplasmic nitrate reductase  NO_3_ → NO_2_ | NAP1  TCTGGACCATGGGCTTCAACCA | 786^b^ | Initial denaturation at 95°C-7min, followed by 37 cycles of denaturation at 95°C-30sec, annealing at 61°C-30sec, extension at 72°C-1min, and final extension at 72°C-4min. |
|  |  |  | NAP2  ACGACGACCGGCCAGCGCAG |  |  |
|  | *nar*G | Respiratory nitrate reductase  NO_3_ → NO_2_ | narG1960f  TAYGTSGGSCARGARAA | 650^c^ | Initial denaturation at 95°C-5min, followed by 8 cycles of denaturation at 94°C-30sec, annealing at X°C-30sec (Touch down PCR -0.5°C per cycle, starting in 54°C and finalizing in 50°C), extension at 72°C-45sec, followed by 30 cycles with fixed annealing at 50°C-30sec, and final extension at 72°C-6min. |
|  |  |  | narG2650r  TTYTCRTACCABGTBGC |  |  |
|  | *nir*S | Nitrite reductase  NO_2_ → NO | nirS1F  CCTAYTGGCCGCCRCART | 890^d^ | Initial denaturation at 95°C-5min, followed by 10 cycles of denaturation at 95°C-30sec, annealing at X°C-40sec (Touch down PCR -0.5°C per cycle, starting in 57°C and finalizing in 52.5°C), extension at 72°C-1min, followed by 30 cycles with fixed annealing at 55°C-40sec, and final extension at 72°C-5min. |
|  |  |  | nirS6R  CGTTGAACTTRCCGGT |  |  |
|  | *nor*B | Nitric oxide reductase  NO → N_2_O | cnorB2F  GACAAGNNNTACTGGTGGT | 389^e^ |  |
|  |  |  | cnorB6R  GAANCCCCANACNCCNGC |  |  |
|  | *nos*Z | Nitrous oxide reductase  N_2_O → N_2_ | nosZ-F-1181  CGCTGTTCXTCGACAGYC | 700^f^ | Initial denaturation at 94°C-3min, followed by 25 cycles of denaturation at 94°C-45sec, annealing at 58°C-60sec, extension at 72°C-2min, and final extension at 72°C-7min. |
|  |  |  | nosZ-R-1880  ATGTGCAKXGCRTGGCAG |  |  |
| Nitrogen Assimilation | *nas*A | Assimilatory nitrate reductase  NO_3_ → NO_2_ | nasA964  CARCCNAAYGCNATGGG | 775^g^ | Initial denaturation at 95°C-5min, followed by 20 cycles of denaturation at 94°C-30sec, annealing at X°C-30sec (Touch down PCR -0.3°C per cycle, starting in 56°C and finalizing in 50°C), extension at 72°C-1min, followed by 10 cycles with fixed annealing at 50°C-30sec, and final extension at 72°C-7min. |
|  |  |  | nasA1735  ATNGTRTGCCAYTGRTC |  |  |

Descriptions for PCR assays can be found in: ^a^Padhi et al. 2017; ^b^Qing et al. 2018; ^c^Philippot et al. 2002; ^d^Braker et al. 1998; ^e^Braker and Tiedje, 2003; ^f^Rich et al. 2003; ^g^Allen et al. 2001

**References**

Alexander, M. (1965). Denitrifying bacteria. In A. G. Norman, & C. A. Black (Ed.), Methods of soil analysis. Chemical and microbiological properties (pp. 1484-1486). Madison, Wisconsin , USA: American Society of Agronomy. doi:https://doi.org/10.2134/agronmonogr9.2.c52

Allen, A. E., Booth, M. G., Frischer, M. E., Verity, P. G., Zehr, J. P., & Zani, S. (2001). Diversity and Detection of Nitrate Assimilation Genes in Marine Bacteria. Applied and Environmental Microbiology, 67(11), 5343-5348. doi:10.1128/AEM.67.11.5343–5348.2001

BIOLOG. (2016, 10 01). BIOLOG GEN III MicroPlates Protocol. Retrieved from Biolog Cell Phenotyping, Microbial Identification to Human Cell Analysis: <https://www.biolog.com/wp-content/uploads/2020/04/00P_185_GEN_III_MicroPlate_IFU.pdf>

Braker, G., & Tiedje, J. M. (2003). Nitric Oxide Reductase (*nor*B) Genes from Pure Cultures and Environmental Samples. Applied and Environmental Microbiology, 69(6), 3476-3483. doi:10.1128/AEM.69.6.3476–3483.2003

Braker, G., Fesefeldt, A., & Witzel, K. P. (1998). Development of PCR Primer Systems for Amplification of Nitrite Reductase Genes (*nir*K and *nir*S) To Detect Denitrifying Bacteria in Environmental Samples. Applied and Environmental Microbiology, 64(10), 3769-3775.

Padhi, S. K., Tripathy, S., Mohanty, S., & Maiti, N. K. (2017). Aerobic and heterotrophic nitrogen removal by *Enterobacter cloacae* CF-S27 with efficient utilization of hydroxylamine. Bioresource Technology, 232, 285-296. doi:https://doi.org/10.1016/j.biortech.2017.02.049

Philippot, L., Piutti, S., Martin-Laurent, F., Hallet, S., & Germon, K. C. (2002). Molecular Analysis of the Nitrate-Reducing Community from Unplanted and Maize-Planted Soils. Applied and Environmental Microbiology, 68(12), 6121-6128. doi: https://doi.org/10.1128/AEM.68.12.6121-6128.2002

Qing, H., DOnde, O. O., Tian, C., Wang, C., Wu, X., Feng, S., Liu, Y., Xiao, B. (2018). Novel heterotrophic nitrogen removal and assimilation characteristic of the newly isolated bacterium *Pseudomonas stutzeri* AD-1. Journal of Bioscience and Bioengineering, 3, 339-345. doi: <https://doi.org/10.1016/j.jbiosc.2018.03.010>

Rich, J. J., Heichen, R. S., Bottomley, P. J., Cromack, K., & Myrold, D. F. (2003). Community Composition and Functioning of Denitrifying Bacteria from Adjacent Meadow and Forest Soils. Applied and Environmental Microbiology, 69(10), 5974-5982. doi: https://doi.org/10.1128/AEM.69.10.5974–5982.2003

Tayeb, L., Ageron, E., Grimont, F., & Grimont, P. A. (2005). Molecular phylogeny of the genus *Pseudomonas* based on *rpo*B sequences and application for the identification of isolates. Research in Microbiology, 156(5-6), 763-773. doi: https://doi.org/10.1016/j.resmic.2005.02.009
